# Influence of birth order, birth weight, colostrum and serum immunoglobulin G on neonatal piglet survival

**DOI:** 10.1186/2049-1891-3-42

**Published:** 2012-12-23

**Authors:** Rafael A Cabrera, Xi Lin, Joy M Campbell, Adam J Moeser, Jack Odle

**Affiliations:** 1 Laboratory of Developmental Nutrition, Department of Animal Science, North Carolina State University, Raleigh, NC, 27695, USA; 2 American Protein Corporation, Ankeny, IA, 50021, USA; 3 Department of Population Health and Pathobiology, College of Veterinary Medicine, North Carolina State University, Raleigh, NC, 27695, USA; 4 Current address: Huvepharma USA, Inc., 525 West Park Drive Suite 230, Peachtree City, GA, 30269, USA; 5 101 Polk Hall, North Carolina State University, Raleigh, NC, 27695, USA

**Keywords:** Birth order, Birth weight, Immunoglobulin G, Colostrum, Survivability

## Abstract

**Background:**

Intake of colostrum after birth is essential to stimulate intestinal growth and function, and to provide systemic immunological protection via absorption of Immunoglobulin G (IgG). The birth order and weight of 745 piglets (from 75 litters) were recorded during a one-week period of farrowing. Only pigs weighing greater than 0.68 kg birth weight were chosen for the trial. Sow colostrum was collected during parturition, and piglets were bled between 48 and 72 hours post-birth. Piglet serum IgG and colostral IgG concentrations were determined by radial immunodiffusion.

**Results:**

Sow parity had a significant (P < 0.001) effect on sow colostral IgG concentration, being 5% higher in multiparous females. Sow colostral IgG concentration explained 6% and piglet birth order accounted for another 4% of the variation observed in piglet serum IgG concentration (P < 0.05); however, birth weight had no detectable effect. Piglet serum IgG concentration had both a linear (P < 0.05) and quadratic effect (P < 0.05) on % survival. Piglets with 1,000 mg/dl serum IgG or less (n=24) had a 67% survival; whereas, piglets with IgG concentrations between 2250 to 2500 mg/dl (n=247) had a 91% survival. Birth order had no detectable effect on survival, but birth weight had a positive linear effect (P < 0.05). Piglets weighing 0.9 kg (n = 107) at birth had a 68% survival rate, and those weighing 1.6 kg (n = 158) had an 89% survival.

**Conclusion:**

We found that the combination of sow colostrum IgG concentration and birth order can account for 10% of the variation of piglet serum IgG concentration and that piglets with less than 1,000 mg/dl IgG serum concentration and weight of 0.9 kg at birth had low survival rate when compared to their larger siblings. The effective management of colostrum uptake in neonatal piglets in the first 24 hrs post-birth may potentially improve survival from birth to weaning.

## Background

Modern swine genotypes have been selected for increased litter size over the last 10–15 years which has resulted in greater heterogeneity of piglet birth weight [1] and decreased pre-weaning survival. Pre-weaning mortality remains unacceptably high with at least 50% of pre-weaning deaths occurring the first 3 days post-birth [2]. Colostrum intake may be low in low birth weight piglets resulting in compromised health and elevated mortality. Many researchers have confirmed that low birth weight piglets grow slower, are fatter, and are more likely to die before weaning [3-5]. Ingestion of colostrum after birth is essential to stimulate intestinal growth and function [6], provide systemic immunological protection via absorption of IgG [7] and it provides energy for thermoregulation [8]. Devillers et al. [9] estimated average piglet colostrum intake to be 300 ± 7 g and sow colostrum yield to be 3.67 ± 0.14 kg. Mersmann [10] showed that the neonatal piglet has lower lipid stores, less gluconeogenic capacity and less phosphorylase activity when compared to other livestock species. Klobasa et al. [11] investigated passive immunity (concentrations of serum immunoglobulins) on 603 neonatal piglets according to birth order, litter size and parity in their first day of life. There was a significant effect of birth order because of the rapid changes in colostrum composition between birth of the first and last piglet of each litter. The effect of birth weight was not detectable in serum IgG concentration and was different for the immunoglobulin classes. There was no significant effect of litter size on passive immunization. Parity had a significant effect on IgG concentration in sow’s colostrum with litter two to six. Machado-Neto and others [12] found that a concentration of IgG of less than 10 mg/ml on postnatal d 1 was associated with an increased pre-weaning mortality in piglets.

Our objective was to examine relationships among birth weight, birth order, serum IgG concentration and their correlation with piglet growth and mortality in a commercial swine facility.

## Methods

### Pig and sow handling

All protocols were carefully supervised and approved by the corporate licensed veterinarians. All standard operating procedures for animal treatment and care were in agreement with published guidelines for animal care [13]. The experimental animals were not subjected to prolonged restraint or surgical procedures and were humanely treated throughout the experiment. The farrowing of 82 sows (Monsanto Choice Genetics) was supervised for a period of one week in a commercial 1800-sow unit during the month of August in Smithfield, NC. All sows farrowed before 116 d of gestation. At farrowing, piglets were dried, birth order recorded, weighed (Model S200 scale, Central City Scale; NE) and ear-tagged (INFECTA + GUARD® Duflex tags, Digital Angel; MN) in both ears. The time of birth of each piglet was recorded. After processing, each piglet was positioned to the sow underline to encourage suckling. We aimed to place 11 piglets on every sow. If a sow birthed more than 11 piglets, the remaining piglets were not enrolled in the study. If a sow had less than 11 piglets, foster piglets were placed to complete 11 but the fostered piglets were not used in the study. Piglets that weighed less than 0.68 kg were not used in the study. Weaning age varied between 16 to 20 days and piglets were weighed again at that time.

### Sow colostrum and piglet blood collection

Sow colostrum was manually collected immediately after sows began farrowing. All teats were sampled into a single composite, and the approximate amount collected per sow was 100 ml. Colostrum was harvested in plastic cups and immediately refrigerated. Piglets were bled between 48 to 72 hours post-birth using a 22 × 1.5” gauge needle and a 6 ml (13 × 100 mm) serum vacutainer tube (Becton Dickinson & Co, Franklin Lakes, NJ). Blood samples were refrigerated and allowed to clot overnight. Serum was collected after centrifugation (10 min × 1300 g, IEC Centra GP8R, DJB Labcare Company, UK) and stored at −20 C until further analysis. Sow colostrum was centrifuged similarly and the defatted fraction containing the IgG was stored at −20 C until further analysis.

### IgG determination

A radial immunodiffusion test was used to determine IgG content in the piglet serum and sow colostrum [14,15]. Measurement of radial immunodiffusion was based on the diffusion of antigen from a circular well radially into a homogenous gel containing specific antiserum for the antigen (in this case, anti porcine IgG). The circle of precipitated antigen-antibody was visualized. The diameter of the precipitation ring was a function of antigen concentration and quantification was based upon comparison to an external standard curve. The radial immunodiffusion plates (Kent Laboratories, Bellingham, WA) contained specific antiserum in agarose gel, 0.1 M phosphate buffer pH 7.0, 0.1% sodium azide as bacteriostatic agent and 1 ug/ml amphotericin B as antifungal agent. Plates contained 0.002 M ehtylenediaminetetracetic acid. After being loaded with 5 μl/well of the serum, the plates are incubated for 24 hours at room temperature.

### Total protein determination

Total protein concentration in the serum samples was determined using the bicinchoninic acid (BCA) protein assay (Thermo Fisher Scientific Inc., Rockford, IL) [16]. Protein concentrations were determined and reported with reference to bovine serum albumin standards. Once the appropriated dilution was determined, the samples were loaded into the wells, incubated for 1 hour and read at 570 nm in a Synergy HT plate reader using KC4™ v3.4 and KC4™ signature software (Bio-Tek Instruments, Inc.; Vermont, USA).

### Statistical analysis

The data were analyzed using the GLM and REG procedures of SAS in order to establish relationships among sow parity, sow IgG colostral concentrations, birth order, and piglet birth weight to piglet plasma IgG and protein concentration. We used a weighed logistic regression analysis in order to determine the effects of pig IgG concentration, piglet birth order and birth weight on % of survival at weaning, with number of piglets per point being the weighting factor. Individual pig was used as the experimental unit.

## Results

**Figure 1:**
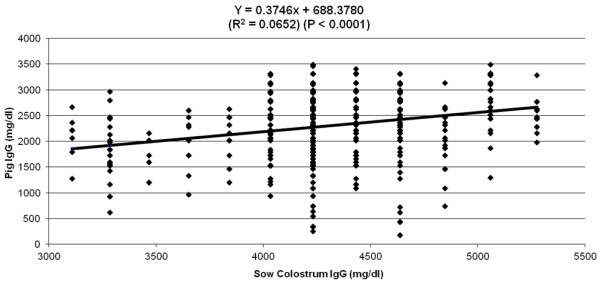
Effect of sow colostrum IgG concentration (mg/dL) collected at the initiation parturition on pig IgG concentration (mg/dL) at 48–72 h after birth.

**Figure 2:**
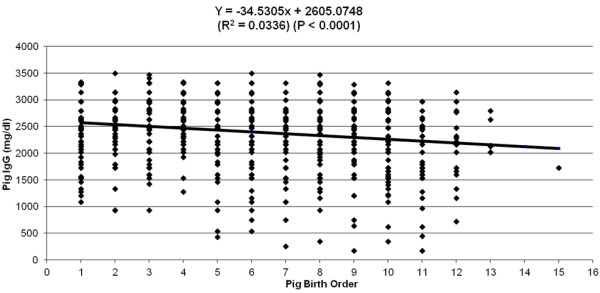
Effect of pig birth order on pig serum IgG concentration (mg/dL) at 48–72 h after birth.

**Figure 3:**
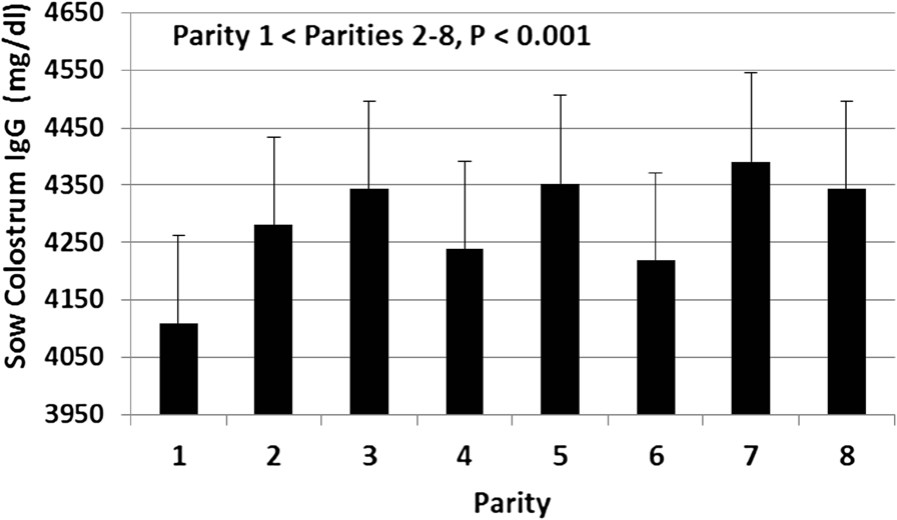
Effect of sow parity on pig IgG concentration (mg/dL) at 48–72 h after birth.

**Figure 4:**
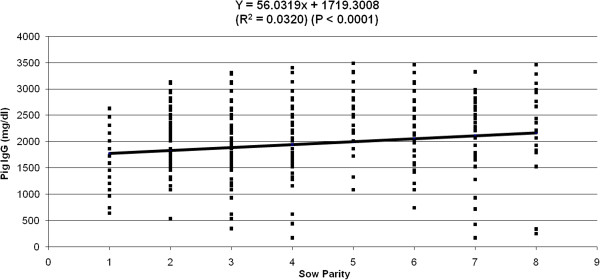
Effect of sow parity on sow colostral IgG concentration (mg/dl) collected at the initiation of parturition.

Sow colostral IgG concentration (Figure 1) explained 6% of the variation observed in piglet serum IgG concentration (P < 0.0001). Piglet birth order and sow parity (Figures 2 and 3) accounted for 4% and 3% respectively of the remaining variation observed in piglet serum IgG concentration (P < 0.0001). However, birth weight had no detectable effect (data not shown) on piglet serum IgG concentration. Sow parity had a significant (P < 0.001) effect on sow colostral IgG concentration (Figure 4). First parity sows had significant lower (P < 0.001) colostral IgG concentration when compared with sows with 2 parities or more. We did not find significant differences in colostral IgG concentrations among sows with 2 parities or more.

**Figure 5:**
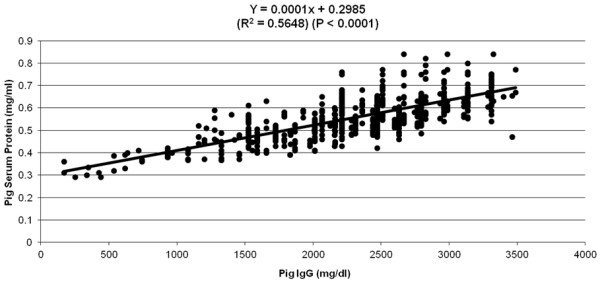
Relationship between pig serum IgG concentration (mg/dL) and pig serum protein concentration (mg/mL) collected at 48–72 h after birth.

Piglet serum IgG concentration was strongly correlated (P < 0.0001) with total serum protein concentration (Figure 5). Piglet birth weight had no detectable effect on piglet blood serum total protein (data not shown).

**Figure 6:**
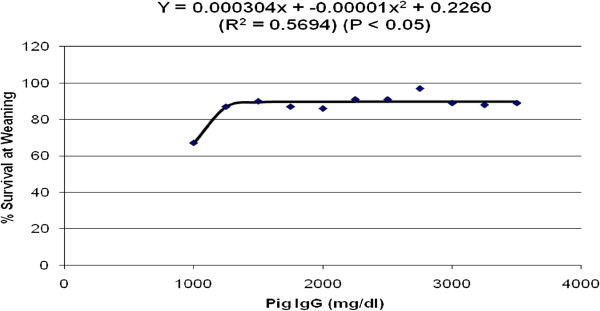
Effect of pig serum IgG concentration (mg/dL) at 48–72 h after birth on piglet survival (%) at weaning.

Piglet serum IgG concentration had both a positive linear and a negative quadratic effect (Figure 6) on % survival at weaning (P < 0.05). Piglets with 1,000 mg/dl IgG or less (n=24) had a 67% survival at weaning; whereas, piglets (n=247) that had serum IgG concentrations between 2250 to 2500 mg/dl had a 91% survival at weaning.

**Figure 7:**
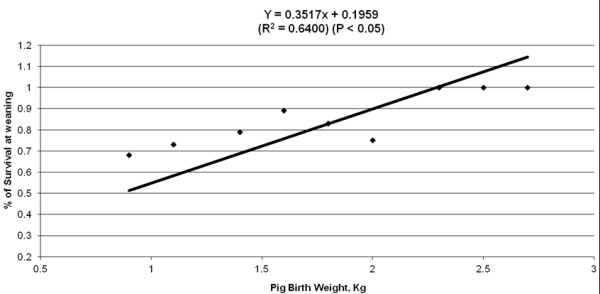
Effect of pig birth weight (kg) piglet survival (%) at weaning.

Birth order had no detectable effect on % survival at weaning (data not shown). Piglet birth weight had a linear effect (P < 0.05) on % survival at weaning (Figure 7). Piglets weighing 0.9 kg (n = 107) at birth had 68% survival rate, and those weighing 1.6 kg (n = 158; ~average birth weight) had an 89% survival.

## Discussion

The most significant finding of this study was that the combined effects of sow parity, sow colostral IgG concentration and birth order only explained 13% of the total variability found in piglet serum IgG concentration. We expected that these factors would have accounted for a greater percentage of the total variability found in piglet serum IgG concentration. Another significant finding was that, contrary to what we would have expected, birth weight had no effect on piglet serum IgG concentration. This lack of effect could be explained by the fact that we physically aided each new born piglet to suckle from their dam immediately after birth.

Piglet serum IgG concentration and birth weight had the greatest effect of any of the variables measured on survival percent at weaning. Stone and Leymaster [17] used a statistical model that fitted the combined effects of serum albumin and birth weight and found that they accounted for 57% of the variation in survival. Hendrix et al. [18] showed that the concentration of IgG in piglet plasma shortly after birth was positively correlated with survival.

We selected piglets with birth weight greater than 0.68 kg because very low birth weight piglets die to a much greater extent than their heavier littermates and this would have limited our blood sampling at 2 d of age. A low-birth weight piglet is disadvantaged when competing with its littermates a) because of its size [7], b) it has a larger surface area when compared to its weight [19,20] and c) it differs physiologically from its littermates and has an increased risk of mortality [21]. Milligan et al. [22] also emphasized that this disadvantage is exacerbated in large litters and litters from older sows. Various researchers [23-25] have argued that low-birth weight piglets lack the ability to successfully extract colostrum from the teats and this reduces their chances of suckling later on, as nursing becomes synchronized.

Blasco et al., [26] reported that 12% of newborn piglets die before weaning. Moreover, more than 50% of the deaths occur in the first 3 d after birth [27] with crushing accounting for 70 to 80% of deaths [28]. Most causes of deaths are due to interactions between the piglet and its environment [29]. Xu et al. [6] attributed these deaths during lactation to the increasing susceptibility to pathogens due to the low immune-competence of the piglets at birth.

We specifically chose IgG as the measuring index of circulating maternal antibodies because it is the most abundant immunoglobulin transferred from the blood to the mammary gland in swine. In sow colostrum, all of the IgG, most of the IgM and 40% of the IgA originate from the maternal blood [30]. Piglets start synthesizing their own IgG after 7 d of age and that amount synthesized depends on the amount of IgG absorbed from colostrum [31]. Our results indicate that the concentration of piglet serum IgG depends on sow colostrum IgG, birth order, and parity. Klobasa et al. [11] investigated passive immunity in 603 neonatal piglets and found that it was affected by birth order, litter size and parity.

We attribute the significant effect of birth order on piglet serum IgG concentration to the rapid changes in colostrum quality and quantity between onset of parturition and last piglet being born in each litter. Not only does the colostral IgG concentration change in the first 24 h of life, but so does other bio-active compounds (growth factors, cytokines and cells) as well [7]. Our results show that the average farrowing time for gilts was 2.4 ± 1.1 hrs, parity 2 was 2.2 ± 0.9 hrs and for parity 3 or more was 2.1 ± 1.2 hrs which is consistent with the farrowing interval reported by others [32-35]. Bourne [34] reported that at six hours post-birth, the protein and immunoglobulin fraction of colostral whey proteins decreased to 50% of the pre-nursing values. It is then safe to conclude that early-born pigs have access to colostrum 50% more concentrated in total protein and immunoglobulins. Harmon et al., [36] reported that late-born piglets had higher mortality than earlier-born littermates. This knowledge of colostrum concentration has led to the development of a swine production practices called “split suckling” in which pigs are removed from their dam at birth to allow early- and late-born piglets to have equal opportunity to acquire high levels of colostral protein.

Klobasa and co-workers [11] also reported that birth weight was only of borderline significance in determining piglet serum IgG concentration. This is also in agreement with our results because we did not detect any relationship between birth weight and piglet serum IgG content.

Machado-Neto et al. [12] showed that lower IgG in colostrum of sows was correlated with lower serum IgG in piglets in the first 20 d postpartum. The levels of piglet serum IgG concentration found in our study agree in general with those reported earlier [37-39]. Thirty-nine percent of the 2–3 d old piglets (n=247) in our study had IgG concentrations between 2250 to 2500 mg/dl, which is a little higher than those reported by Machado-Neto et al. [12] by d 2 (2470 mg/dl) and d 3 (1940 mg/dl). This might be attributed to the fact that we carefully dried the piglets immediately after birth and we physically aided each new born piglet to suckle from their dam immediately after the neonatal was born. This observation is quite important to disclose because Blecha and Kelly [37] reported that a single 2.5-hr exposure to cold air (15°C) temperature at birth reduces the subsequent acquisition of colostral immunoglobulin.

We hypothesized that piglet serum IgG concentration is highly correlated with pre-weaning mortality. We found both a linear and quadratic response when correlating piglet serum IgG concentration and piglet survival at weaning. Logistic regression was shown to be an appropriate and useful technique for analysis of factors affecting piglet survival. The advantages of logistic regression are that it can be applied to continuous as well as discrete predictor variables and the fitted regression coefficients are readily translated into the odds ratio, giving a mathematical quantification to observed differences [38]. We found that when piglets had serum IgG concentration of 1,000 mg/dl, they had a 67% chance of survival. Machado-Neto et al. [12] found that a concentration of IgG of less than 10 mg/ml on postnatal d 1 has been associated with an increased pre-weaning mortality in piglets. We cautioned that the minimum level of serum immunoglobulin that should be achieved to ensure survival of piglets may depend upon the environment, farm management, seasons and disease conditions. Blecha and Kelly [37] reported that live born piglets that die before 21 days of age had lower immunoglobulin concentrations in serum during the first day of life than did piglets that live. These results are consistent with those reported by Hendrix et al. [18] indicating that piglets that survived to 21 days of age had a higher concentration of immunoglobulin, shorter birth interval, heavier birth weight and were born earlier in the litter than those piglets that were born alive but died before 21 days of age. Piglets from litters with high mortality rates show weight loss, do not synchronize in suckling, fight more and for longer periods and have lower IgG levels, indicating problems in the sow [39,40].

We argue that while systemic serum IgG concentration might indicate an animals’ general immune status, circulating immunoglobulin content gives no indication as to the specificity of the immunity that may be present or that may have developed in the tissue immune system (e.g. IgA secreted in the intestinal tract), which is important in resisting certain diseases common in young piglets. We recognized that colostrum contains others substances (i.e. energy) besides immunoglobulins that also are important for piglet survival. Finally, a reduced consumption of immunoglobulins may predispose piglets to selected kinds of infectious diseases.

The reason we placed 11 pigs per sow was due to the interaction between the number of piglets in a litter and the stimulation they provide to sow’s milk output. When there are fewer piglets suckling, the longer the pre-ejection massaging needed to cause a milk ejection [41]. The weight of the piglets at birth plays an important role in stimulating the sow to produce milk. A large piglet may perform the massage of its teat before ejection more vigorously, thus achieving a greater blood flow to the teat and thereby bringing more of the limited supply of oxytocin to its own teat [42]. It is well known that suckling leads to an activation of neurohormonal reflexes that result in the release of oxytocin [43], prolactin [44], gut hormones such as gastrin, somatotropin and vasoactive intestinal polypeptide (VIP) and pancreatic hormones such as insulin and glucagon [45-48].

Birth weight has been widely known to be a very important economic trait in swine production. Birth weight drives pre- and post-weaning piglets’ growth. Piglets’ ability to have high colostrum intake is determined by their body weight at birth, which is also the main factor of their vitality and their ability to stimulate the udder to extract colostrum [9]. Our results showing that low birth weight in piglets correlates with decrease survival and lower post-natal growth rates are consistent the work reported by others [49-51]. Fix et al. [3] found that low birth weight piglets grew slower, were fatter and were more likely to die at weaning. Beaulieu et al. [52] found that lighter birth weight piglets had reduced BW at weaning, 5 and 7 wk post-weaning, and at first pull and had increased days to market. Rehfeldt and Kuhn [53] argued that in the majority of low-birth weight piglets low numbers of muscle fibers differentiate during prenatal myogenesis, for genetic or maternal reasons, and those low-birth weight piglets with reduced fiber numbers are unable to exhibit postnatal catch-up growth. Prenatal development is mainly dependent on a close interrelation between nutritional supply/use and regulation by hormones and growth factors. They found that low-birth weight piglets showed the lowest growth performance and the lowest lean percentage at slaughter.

## Conclusions

We found that sow colostrum, pig birth order and sow parity collectively had a small impact on piglet serum IgG concentration and piglet birth weight did not. Piglet serum IgG concentration was highly correlated with piglet serum total protein concentration. Piglet serum IgG concentration and birth weight had the greatest effect of any of the variables measured on % survival at weaning. Collectively, these data indicate that pigs with ≤ 1,000 mg/dl IgG at 2–3 days of age have reduced survivability and may benefit from IgG supplementation early in life. The number of piglets in this category was low in this study (24/637= 3.8%) but this number is biased downward because we excluded pigs weighing less than 0.68 kg from the experiment. If an IgG-rich supplement were directed to low birth weight piglets, piglets of high birth order and/or piglets from low-parity sows, survivability may be improved. In addition to IgG, the role of colostrum’s bioactive compounds such as cells and growth factors in the development of the immune system merits further investigation.

## Abbreviations

IgG: Immunoglobulin G; IgA: Immunoglobulin A; SAS: Statistical Analysis System; GLM: General Linear Model; REG: Regression; Mg: Milligram; Dl: Deciliter; Kg: Kilograms; Ml: Milliliter; Hr: Hour; FASS: Federation of Animal Science Societies; ID: Identification; CHG: Chlorhexidine gluconate; C: Celsius; M: Molar; BCA: Bicinchoninic Acid; BSA: Bovine Serum Albumin; VIP: Vasoactive Intestinal Polypeptide; BW: Body Weight; ADG: Average Daily Gain.

## Competing interests

Full financial support for this trial was provided by American Protein Corporation (APC), Ankeny, IA 50021. Dr. Joy Campbell (one of the author in this manuscript) serves as Director of Research and Development for American Protein Corporation. APC is the world’s largest producer of functional proteins. Functional proteins are a complex mixture of biologically active proteins that help support and maintain normal immune function. APC’s functional proteins are derived from unique components found in bovine and porcine blood, and are consumed by millions of animals around the globe. No products from APC were used in this trial.

## Authors’ contributions

RAC as the lead author was in charge of designing the research protocol, obtaining all materials needed for it, executing it, carrying out all the lab work, data collection and analysis and finally writing the manuscript. XL was instrumental on guiding the lead author on the statistical analysis and lab methods. JC gave significant contribution on the importance of colostrum and immunoglobulins and their effects on neonatal survival. She assisted in designing the study and provided excellent suggestions to the final version of the manuscript. JO as a co-advisor to the lead author, he was involved in the design, execution and interpretation of the results. He also assisted with blood collection and statistical analysis. AJM as a co-advisor to the lead author, he was involved in the design and interpretation of the results. All authors read and approved the final version of the manuscript.

## Authors’ information

RC holds a PhD in Animal Nutrition from North Carolina State University (NCSU). His area of research is neonatal survival, nutrient digestibility and gastrointestinal health of swine. In 2001, he was awarded the “Innovative Award Applied Research” by National Pork Producer Council (NPPC) at the Midwest Animal Science Meeting in Des Moines, Iowa. He is a member of the North Carolina Pork Council and the American Society of Animal Science. He currently serves as Director of Swine Technical Services for Huvepharma USA, Inc. XL holds a PhD in Animal Nutrition from China Agricultural University. His main areas of research are neonatal survival and lipid metabolism primarily focused on the regulation of fatty acid oxidation during neonatal development and epigenetic regulation of fetal development and placenta growth. He is also interested in the role of polyunsaturated fatty acid in the development of neonates. He is a Research Assistant professor at the Department of Animal Science at NCSU and a member of American Society of Animal Science. JC has a PhD in Nutritional Sciences from the University of Illinois Urbana-Champaign. She works with functional proteins (such as plasma) and their impact on gastrointestinal health. Her main research interests are Nutrition and Animal Health. She serves as Director of Research and Development North America for American Proteins Corporation (APC) in Ankeny, IA and is a member of American Society of Animal Science. She received the ASAS/ADSA Outstanding Young Agribusiness Award from American Society of Animal Science. AJM holds a MS in Swine Nutrition, a PhD in Gastrointestinal Physiology and a Doctor of Veterinary Medicine (DVM) all from NCSU. His main area of research is to study basic mechanisms of stress-induced intestinal dysfunction. Stress is an important contributing factor to enteric disorders of veterinary species and humans however, the mechanisms are poorly understood. His work has focused on the role of mucosal mast cells in psychological stress-induced disturbances in intestinal mucosal barrier function. He believes that this work will have important implications in the understanding of stress-related gut disorders such as infectious diarrhea, Inflammatory Bowel Disease, and Irritable Bowel Syndrome, and will facilitate the design of novel preventative and treatment strategies for veterinary and human patients suffering from these disorders. He is an assistant professor of GI physiology and swine medicine at NC State College of Veterinary Medicine. He is member of several professional societies including the American Physiological Society, American Association of Swine Veterinarians, and American Gastroenterological Association (AGA). He has over 34 peer-reviewed publication focused mainly in the area of gastrointestinal health in swine. JO has a PhD in Nutritional Biochemistry from the University of Wisconsin. As a Williams Neal Reynolds Professor in the Department of Animal Science at NCSU, his research interests are molecular and metabolic regulation of lipid digestion and metabolism; neonatal nutrition; intestinal growth and metabolism in normal and pathophysiological states. His program is focused on using the young piglet as a model for the human infant in nutrition and digestive physiology. He also has teaching responsibilities in the areas of nutrition and biochemistry. His most recent awards include “Williams Neal Reynolds Distinguished Professor” and “The Outstanding Graduate Instructor” both given by the College of Agriculture and Life Science at North Carolina State University, the “Animal Growth and Development Research” given by the American Society of Animal Science. He was a member of the National Research Council (NRC) committee which recently published the new 2012 Nutrient Requirement of Swine. He is an Associate Editor in Advances in Nutrition (American Society for Nutrition) and the Journal of Animal Science and Biotechnology.
